# An Intervention Target for Myocardial Fibrosis: Autophagy

**DOI:** 10.1155/2018/6215916

**Published:** 2018-04-02

**Authors:** Chunmiao Lu, Yusong Yang, Yaping Zhu, Shichao Lv, Junping Zhang

**Affiliations:** ^1^Graduate School, Tianjin University of Traditional Chinese Medicine, Tianjin 300193, China; ^2^Department of Cardiovascular Medicine, First Teaching Hospital of Tianjin University of Traditional Chinese Medicine, Tianjin 300193, China; ^3^Department of Geriatric Medicine, First Teaching Hospital of Tianjin University of Traditional Chinese Medicine, Tianjin 300193, China

## Abstract

Myocardial fibrosis (MF) is the result of metabolic imbalance of collagen synthesis and metabolism, which is widespread in various cardiovascular diseases. Autophagy is a lysosomal degradation pathway which is highly conserved. In recent years, research on autophagy has been increasing and the researchers have also become cumulatively aware of the specified association between autophagy and MF. This review highlights the role of autophagy in MF and the potential effects through the administration of medicine.

## 1. Myocardial Fibrosis

MF is caused by various factors, which lead to excessive deposition of collagen fibers, significant augmentation of collagen concentration and volume fraction, and disproportion and permutation disorder of collagen types in the myocardium [1]. MF can be generally categorized into two types: replacement and interstitial fibrosis [2]. The former one is also referred to as repairable fibrosis, which is often caused by myocardial necrosis. Conditions related to replacement fibrosis include myocarditis, myocardial infarction, hypertrophic cardiomyopathy, and toxic cardiomyopathies [3]. The later one has two main subtypes, reactive and infiltrative interstitial fibrosis. Reactive fibrosis mainly exists in aging and hypertension. Infiltrative interstitial fibrosis, however, is rather rare, which results from progressive deposition of glycosphingolipids or insoluble proteins in the interstitial space [4]. Accumulating research has demonstrated that there is a close and complex relationship between MF and cardiovascular diseases, which is represented by the following listed diseases: ischemic cardiomyopathy, viral myocarditis, hypertensive heart disease, diabetic cardiomyopathy (DCM), hypertrophic cardiomyopathy, and so on [5–8]. MF is the principal pathological basis of cardiac remodeling, which is also a potential risk factor for cardiovascular events such as arrhythmias, heart failure, and sudden cardiac death. The rennin-angiotensin-aldosterone system (RAAS), cytokines, chemokines, endothelin-1, reactive oxygen species, and platelet-derived growth factors are closely associated with MF [9]. However, in recent years, with the increasing research on autophagy, more and more studies have shown that autophagy plays an important role in the process of MF [10]. Even if it is still unclear whether autophagy has promoting or suppressing effects in MF, regulation of autophagy might become one of the potential targets for the treatment of MF.

## 2. Molecular Mechanisms of Autophagy

Autophagy is a complex and evolutionary conserved catabolic system; unnecessary or dysfunctional or long-lived proteins and cellular components are degraded in lysosomal machinery, which is essential for maintaining the normal structure and function of cells [11]. Autophagy is not only a widespread normal physiological process in vivo, but also thought to be a defense when cells resist the adverse environment. In addition, it also participates in the pathological process of a lot of diseases [12]. Induction and regulation of autophagy are very complicated processes. As a major cytoprotective mechanism, although autophagy maintains dynamic homeostasis under regular conditions, many stimulating factors can induce cell autophagy, such as hypoxia [13], starvation [14], oxidative stress [15], ischemia-reperfusion (I/R) [16], pathogen infection [17], and endoplasmic reticulum stress [18]. Depending on the different ways in which the cell material is transported to the lysosomes, autophagy can be categorized into macroautophagy, microautophagy, and chaperone-controlled autophagy [19]. During macroautophagy, soluble proteins and cell organelles which have been dysfunctional or necrotic in the cytoplasm are surrounded by double-membrane autophagosomes and then transported to lysosomes for degradation. It is the most common and significant way of the autophagy, which will be discussed in detail in the following content. Microautophagy sequesters and engulfs the various cytoplasmic constituents via direct engulfing by the invaginations of the lysosome membrane [20]. Additionally, chaperone-controlled autophagy is a selective process; cytosolic substrate proteins and a KFERQ-like motif are recognized as chaperones, which then unfold and transport into the lysosome [21].

Macroautophagy is prevalent, which is referred to hereafter as autophagy. Accordingly, the process of autophagy can be distributed into different steps including induction, the autophagosome precursor nucleation, autophagosome expansion, fusing with the lysosome, and moreover recycling of the degraded cargo [36, 37]. Autophagy induction and nucleation: the mammalian target of rapamycin complex 1 (mTORC1) is a signaling pathway that plays an irreplaceable role. Autophagy induction is mainly through interaction between mTORC1 and formation of the Unc-51-Like Kinase (ULK) complex which is composed with ULK, FAK-Family Interacting Protein of 200 kDa (FIP200), and Autophagy-related Protein 13 (Atg13) [38, 39]. Nucleation refers to how Activating Molecule in Beclin-1-Regulated Autophagy (AMBRA1) is phosphorylated by the ULK complex and then activates the phosphatidylinositol-3-kinase (PI3K) complex which is composed of Beclin-1, AMBRA1, Vacuolar Protein Sorting 15 (VPS15) and VPS34, the regulatory kinase VPS15, Atg14, Atg38, and VPS30/Atg6, which are all necessary for nucleation [40, 41]. The sign at this stage is phosphorylated Beclin-1. Phagophore expansion: this step depends on two major ubiquitin-like (Ubl) conjugation systems including the Ubl proteins Atg12 and Atg8 [42, 43]. Autophagosome fusion refers to fusion of autophagosome and lysosome to originate autolysosome. This process is regulated by cytoskeleton, endosomal sorting complex required for transport (ESCRT) and Rab GTPases. Cargo degradation and recycling: once the autolysosome forms, the autophagic body membrane is quickly degraded by a putative lipase and the inner cargoes are degraded by resident hydrolases. The resulting macromolecules such as amino acids are released back into the cytoplasm for recycling [44].

One basic point is that autophagy is a highly dynamic and multistep process, just like other cell pathways, so it could be positive regulation or negative regulation in many steps. Autophagy deficiency leads to abnormal accumulation of proteins and organelles, but excessive autophagy leads to autophagic cell death. Autophagy is common in many cardiovascular diseases and it is closely related to MF. So many studies pointed out that the activity of autophagy needs to stay at an optimal level to preserve normal cardiovascular function [45].

## 3. The Role of Autophagy in Myocardial Fibrosis

### 3.1. Pressure Overload-Induced Cardiac Disease

Under the condition of long time pressure overload, the heart would have a series of pathological changes including cardiomyocyte hypertrophy and interstitial fibrosis. Autophagy participates in the pathogenesis of heart diseases induced by pressure overload, during which the heart changes from the beginning of the compensatory phase to the late decompensation [46]. Zhang et al. reported experimental hypertensive swine model that was established with 10 weeks of unilateral renovascular hypertension. Ten domestic pigs were classified as mild or moderate hypertension (above or below 75%); meanwhile seven normal pigs served as controls. The results showed in moderate hypertension the Beclin-1, Atg12–5, and microtubule-associated protein-1 light-chain (LC)3-II/LC3-I ratio were increased, but in mild hypertension they were not. The increase of autophagy inhibitors of Akt and the effector pS6 in moderate hypertension was lower than that in mild hypertension, although both groups increased. It was clearly demonstrated that, during the exacerbation of left ventricular hypertrophy (LVH), autophagic activity is stimulated, and the activity level of autophagy is also different in different degrees of disease [47]. Accumulating research has shown that autophagy plays a key role in MF of pressure overload-induced cardiac disease, although the precise mechanism remains unclear and controversial. A study has found that the overexpression of metallothionein mice and wild virus B mice was induced experimental hypertension model; mouse was given the autophagy inhibitor 3-methyladenine (3-MA) (10 mg/kg/days, i.p.) for 14 days. Protein level of autophagy marker p62 was increased and autophagy signaling molecules of p70s6 K and mTOR were activated by  _L_-NAME. The beneficial effect of metallothionein against  _L_-NAME was nullified, which appeared to result from the autophagy inhibition. So it was possible that metallothionein protected against  _L_-NAME-induced myocardial anomalies through restoration of autophagy [48]. Another study suggested that berberine could effectively attenuate left ventricular remodeling through an autophagy-dependent mechanism in cardiac hypertrophy rat models induced by transverse aortic contraction (TAC). The upstream signaling of autophagy including extracellular signal-regulated kinase (ERK1/2), the mTOR, and p38 mitogen-activated protein kinase (MAPK) phosphorylation were significantly inhibited by berberine. In certain cases, enhancing autophagy has a beneficial effect on anti-MF [49]. Excessive activation of angiotensin II type 1 receptor (AT1R) could lead to myocardial fibrosis and cardiac hypertrophy. Previous studies have shown that myocardial remodeling was suppressed with the inhibition of AT1R [50, 51]. Lin et al. cultured cardiomyocytes of neonatal rats which were induced by TAC. After having received a 48-hour mechanical stretch, the cardiomyocytes were assessed regarding autophagic marker LC3b-II. The results indicated that the AT1R activated by mechanical stretch could mediate activation of p38MAP kinase for inducing cardiomyocyte autophagy and the consequences of exacerbation of ventricular remodeling [52]. Additionally, setting a model of C57BL/6 mice induced by TAC might lead to cardiac hypertrophy. It also used siRNA for protein kinase D (PKD) knockdown. Treatments with PKD siRNA significantly ameliorated the cardiac hypertrophy and fibrosis by promoting cardiac autophagy via downregulated AKT/mTOR signaling pathway [53]. However, in the face of pressure overload, there are some reports showing diminished cardiac autophagy or autophagy flux to the compromised cardiac function [54]. So it is worth noting that more research suggested autophagy plays a detrimental role in pressure overload-induced cardiac disease. A study confirmed that Calhex 231 could ameliorate myocardial hypertrophy induced by angiotensin II (Ang II) via inhibiting autophagy though the LC3-II/LC3-I ratio was significantly reduced in Ang II-induced cardiomyocyte hypertrophy model [31]. Studies have shown that aliskiren ameliorated heart hypertrophy and fibrosis induced by pressure overload. In that study, expression of LC3-II and Beclin-1 proteins and Atg5 and Atg16 L1 mRNAs was increased after establishing the model. Administration of aliskiren could ameliorate or block the above pathological alterations, which suppress AngII-PKC*β*I-ERK1/2-regulated autophagy [30]. Recent studies revealed that, during the development of myocardial hypertrophy, the level of autophagy decreased while the level of autophagy in the heart failure significantly increased [55, 56]. It is puzzling that the exact roles of autophagy are not clear in cardiac hypertrophy and fibrosis especially in its transition to heart failure [53]. At various stages of either the cardiac hypertrophy or heart failure, timing is one of the key factors for the different roles of autophagy.

### 3.2. Myocardial Ischemia/Reperfusion

Ischemic heart disease is one of the most common cardiovascular diseases. Although timely perfusion can effectively reduce total mortality, the problem with restoring blood flow is reperfusion injury. So far, accumulating research has proved that autophagy is closely related to MF of I/R. However, the mechanism of autophagy in myocardial I/R injury is unclear and controversial [57]. A study showed that 8-week-old mice were infused with recombinant cellular repressor of E1A-stimulated genes (CREG) protein for 7 days. Time to build the model: the mice have gone through myocardial ischemia for 30 min and reperfusion for 24 h. The result showed that lysosomal autophagy activated by human CREG could protect the heart against myocardial I/R injury-induced cardiomyocytes apoptosis [58]. Another study found that autophagy might protect cardiomyocyte against apoptosis through the clearance of CLP36 in Atg7-deficient mice [57]. In contrast, there is some research that suggests that autophagy has a negative effect on myocardial I/R. Establishment of an in vitro model of simulated I/R by using human umbilical vein endothelial cells: the study showed that I/R promotes the activity of NF-*κ*B p65 mediated Beclin 1-associated autophagic flux, thereby exacerbating myocardial injury. These results suggested the possibility that inhibition of excessive autophagy could prevent I/R-induced myocardial injury and anti-MF [59]. The conclusion is that the inhibition of excessive autophagy is beneficial to the heart and could also be obtained in in vivo experiment, as Jian et al. showed in their study [60]. One study also has shown that a basic fibroblast growth factor (bFGF) increased the survival of cardiomyocytes through the PI3K/Akt/mTOR pathway in the myocardial I/R model. During the early stage of I/R, autophagy was excessively activated, and the treatment of bFGF stimulated phosphorylation of PI3K/Akt then further enhanced mTOR signaling to result in autophagy inhibition. It indicated that the role of bFGF is related to inhibiting excessive autophagy [61]. In addition, in a study about H9c2 cell model of oxygen-glucose deprivation (OGD) and reperfusion, the results showed that after OGD 12 h treatments and reperfusion for 6 h the expression of thioredoxin-2 (Trx2) was obviously decreased. The data indicated that Trx2 inhibits autophagy and apoptosis to protect cardiomyocytes [62]. In summary, autophagy in myocardial I/R is the reason why such a large difference occurs, because most studies are now focused on single myocardial I/R, but do not assess the function of autophagy in myocardial I/R state compared to different studies.

### 3.3. Diabetic Cardiomyopathy

Interstitial and/or perivascular fibrosis is a structural hallmark in DCM [63]. Under pathological conditions, such as being exposed to oxidative stress, hyperglycemia, and dyslipidemia, fibroblasts are stimulated by external discomfort, triggering autophagy [64]. Gao et al. fed rats with a high-fat diet with streptozotocin (STZ) to set the diabetic model and sacrificed them after 1, 6, and 7 months. Beclin-1 and the expression of collagen types I and III were upregulated and protein LC3 II/I ratio was increased while the content of P62 was decreased, and as time went by all the changes were aggravated. The results indicated that autophagy exists in diabetic MF, and it changes over time [65]. A study showed that 1,25-dihydroxyvitamin-D3 (1,25D3) partly attenuated the interstitial fibrosis and myocardial hypertrophy by enhancing autophagy in type 1 diabetic rats induced by STZ [27]. On the contrary, accumulating research has reported that autophagy levels decreased in DCM [66]. Another study suggested that diminished autophagy was a beneficial adaptive response for reducing interstitial fibrosis, whereas cardiac damage exacerbated through autophagy was restored [67]. To investigate the role of autophagy in type 2 diabetic mellitus, zhang et al. found that Zucker diabetic fatty rats were given liraglutide, AMP-activated protein kinase (AMPK) phosphorylation was increased, mTOR phosphorylation was decreased, and MF was alleviated; the data provided important evidence that liraglutide mediated diabetic fibrosis by promoting AMPK-dependent autophagy [26]. In another study of type 2 diabetes mellitus, the researchers found that trimetazidine treatment ameliorated insulin resistance and reduced cardiomyocyte apoptosis with restoring cardiac autophagy. It was also thought to be associated with AMPK-dependent pathway; trimetazidine inhibited phosphorylation of P38 MAPK and ERK to reduce MF [25]. At present, many studies on the relationship between MF and autophagy in diabetes have shown that it is related to AMPK. AMPK can upregulate autophagy. In diabetes, the activity of AMPK was inhibited, and autophagy mediated by AMPK-dependent pathway also declined, then leading to heart damage such as MF. Diabetic mice under the chronic administration of metformin, Beclin-1, and Bcl-2 were dissociated, and AMPK-induced autophagy was activated [68]. The change of autophagy level is correlated with cardiac fibrosis, which means in diabetic cardiac fibrosis there is a potential synergistic role of cardiac autophagy [65].

### 3.4. Cardiac Aging

The heart is extremely sensitive to the aging process and cardiac aging is an irreversible biological process [69, 70]. There are many well-acknowledged features of cardiac aging, such as hypertrophic and cardiac fibrosis [71]. Increasing evidence suggests that autophagy is intimately related to cardiac aging [72]. Unfortunately, the activity of autophagy declines in cardiomyocytes with age [73]. A study showed that the same C57/Bl6 mice were assessed by serial echocardiography every 3 months at 18 months of age. These aging animals had fibrosis and LVH. The level of caspase-3 was activated at an early stage of cardiac dysfunction, and relatively high levels of autophagy and antiapoptotic factors existed in the aging hearts at that time [74]. Another study reported that calstabin-2 knockout mice were compared to control wild-type mice, cell cycle inhibitors p16 and p19 were increased, cardiac fibrosis was augmented, and cell death occurred in calstabin-2 knockout mice. The results demonstrated that calstabin-2 deletion caused AKT phosphorylation, augmented mTOR activity, and damaged autophagy [75]. In addition, cardiomyocytes have high reliance on mitochondrial energy metabolism, but mitochondrial autophagy was impaired in cardiac aging. Transgenic mice, regarding cardiac specific expression of the heat shock protein 27, demonstrated less interstitial fibrosis and low accumulation of LC3-II and p62 in old hearts. Furthermore, levels of Atg13, Vps34, small GTPase Rab7, PTEN induced putative kinase 1 (PINK1), and Parkin were higher in old transgenic hearts than old wild-type hearts [76]. Mitophagy activation could inhibit myocardial fibrosis in cardiac aging.

## 4. Potential Effect of Autophagy in the Treatment of Myocardial Fibrosis

MF widely exists in many cardiovascular diseases including hypertension, myocardial ischemia, diabetic cardiomyopathy, and heart failure [9]. So far, however, no effective drugs are available to treat MF. In fact, accumulating study has paid great attention to drugs on anti-MF therapy through in vitro and in vivo experiments. Studies have shown that some drugs or plant extracts can inhibit MF through autophagy regulation, as well as reducing myocardial damage and improving cardiac function [22, 77, 78]. Promoting or suppressing autophagy all can achieve the purpose of inhibiting MF and ameliorating heart function, respectively, concluded from different studies. A study showed that atorvastatin could improve survival of cardiomyocytes and reduce fibrosis and infarcted area of acute myocardial infarction (AMI) rat model. The expression of autophagic protein LC3 and the AMPK/mTOR activity were increased and induction of proapoptotic protein Bax was inhibited, which could be reversed by AMPK inhibitor Compound C. So atorvastatin maybe induced autophagy via AMPK/mTOR pathway [22]. Another study on the regulation of autophagy by atorvastatin was under the condition of spontaneously hypertensive rats model. Spontaneously hypertensive rats were treated by atorvastatin for 6 or 12 months. Wistar-Kyoto rats were used as control group. Some biomarkers of cardiac autophagy activating were elevated. It came to the similar conclusion that atorvastatin increased cardiac autophagy through the Akt/mTOR pathway [23]. Moreover, in pressure overload mice model induced by aortic banding (AB), mice strictly received cucurbitacin B treatment (gavage, 0.2 mg/kg/2 day) after 1 week of surgery. And after 4 weeks of surgery, cucurbitacin B demonstrated a strong antifibrosis and a antihypertrophy ability. The cucurbitacin B mediated that significantly mitigated cardiac hypertrophy could be attributable to the autophagy increase. It was related to the blockade of Akt/mTOR/FoxO3a pathway [24]. In addition, MF is a prominent pathological characteristic in diabetic myocardial damage [32]. Anti-MF therapy is essential in DCM. Setting type 2 DCM rat model by low-dose STZ and high-fat diet, the results suggested that early administration of trimetazidine inhibited phosphorylation of P38 MAPK and ERK to effectively reduce MF. Cardiac autophagy was restored, insulin resistance and metabolic disturbance were ameliorated, and cardiomyocyte apoptosis was reduced [25]. Furthermore, liraglutide has been demonstrated to have the effect of attenuation of myocardial damage through promoting autophagy in Zucker diabetic fatty rat [26]. Some research found that diabetic patients with vitamin D deficiency have a high prevalence of cardiac hypertrophy and fibrosis. It may be a feasible method to treat diabetic cardiomyopathy through vitamin D supplementation. Surely, in STZ-induced diabetic rats and high-glucose cultured H9C2 cells, 1,25D3 could increase autophagy via the *β*-catenin/T-cell factor 4 (TCF4)/glycogen synthase kinase-3*β* (GSK-3*β*) and mTOR signaling pathway. It partly attenuated the interstitial fibrosis and myocardial hypertrophy [27]. Research from Eisenberg et al. was a model of physiological cardiac aging mice, and it received the natural polyamine spermidine intervention. This result showed that spermidine could reduce cardiac fibrosis, preserve diastolic function, and exert cardioprotective effects, which was accompanied by mitophagy, mitochondrial respiration, and cardiac autophagy enhancement. And the augmentation of titin phosphorylation was also taken into account [28]. Then this leads to the discussion of spermidine promoting cardioprotective autophagy [79]. Therefore spermidine-enriched diets may become a feasible strategy for preventing cardiovascular disease in the future.

Nevertheless, in addition to improving autophagy, there are a number of studies that indicate that drugs or plant extracts treat MF by reducing the level of autophagy. Recently, a study reported that isoflurane protected cardiomyocytes following I/R injury, even if it is perennially used as a common inhaled anesthetic. Isoflurane could reduce the expression of nucleotide binding oligomerization domain containing 2 (NOD2), and overexpression of NOD2 is always accompanied by the expression of autophagy-related genes increasing; then isoflurane decreased autophagosome generation [29]. Weng et al. assessed anti-MF of aliskiren in the heart pressure overload mice induced by TAC. Aliskiren and the autophagy inhibitor were administered to the mice for 4 weeks altogether. Aliskiren significantly suppressed the high levels of Ang II, phosphorylation of PKC*β*I/*α* and ERK1/2, and the expressions of LC3-II and Beclin-1 proteins as well as Atg5 and Atg16 L1 mRNAs, which showed aliskiren could inhibit Ang II-PKC*β*I-ERK1/2-regulated autophagy [30]. Another research showed that Calhex 231 could ameliorate MF and myocardial hypertrophy via inhibiting the expression of CaSR and autophagy in pressure overload or Ang II-induced rat model [31]. In also a study of diabetic cardiomyopathy, xiao et al. investigated the predominant effect of hydrogen sulfide on the continuous progression of diabetes-induced MF. By contrast with that mentioned above, hydrogen sulfide attenuated autophagy via the PI3K/AKT1 signaling pathway to ameliorate MF [32]. Chloroquine, an autophagy inhibitor, could ameliorate cardiac diastolic function and decrease the cardiomyocyte apoptosis, autophagolysosomes, and cardiac fibrosis in STZ-induced diabetic mice [33].

As mentioned above, insufficient autophagy leads to abnormal accumulation of proteins and organelles in the myocardium, while excessive autophagy can lead to overdigestion and degradation of cardiac proteins and organelles. It is very important to achieve autophagy balance in cardiomyocytes [34]. More and more researchers are paying attention to the balance of autophagy, not just increasing or decreasing the level of autophagy. There was a study that reported that the left main coronary artery of rats was ligated for 1 h and then reperfused for 3 h. Administrating of phellinus linteus mycelium could alleviate myocardial damage through autophagic regulation, which the accompanying evidence was that caspase-3 and caspase-9 activation decreased, Bcl-2/Bax ratio increased, AMPK phosphorylation elevated, and Beclin-1 and p62 levels decreased. This study emphasized the enhancement of protective autophagy and inhibition of excessive autophagy to achieve autophagy balance [35]. The articles of this section are summarized in Table 1.

## 5. Conclusions

There has been a lot of evidence indicating that autophagy plays a significant role in the occurrence and progression of MF. Nevertheless, it remains unclear whether autophagy has a negative or positive effect in different types of MF and different stages of the disease, and the mechanism is worth further exploration. Because of the difference between experimental design and conditions, in the study of MF under the same disease, the conclusions are different or even, on the contrary, we can only refer to them as reference. Certainly, keeping autophagy balance has been recognized. Furthermore, achieving the purpose of anti-MF through the administration of drugs, some drugs can treat different types of MF while the same kind of MF can be regulated by many drugs. The autophagy, MF, and the relationship between both need intensive study.

## Figures and Tables

**Table 1:** Potential effect of autophagy in the treatment of myocardial fibrosis.

Medicine	Disease	Cells/tissues	Effects	Mechanisms	Pathway	Reference
Atorvastatin	AMI	Rat	Autophagy ↑	Ejection fraction, LC3 ↑ Fibrosis, infarcted area, inflammatory level, Bax ↓	AMPK/mTOR pathway	[22]

Atorvastatin	Hypertension-induced cardiac hypertrophy	SHRs	Autophagy ↑	Expression of protein-1 light chain 3-II and Beclin-1 protein ↑ Akt and mTOR expression ↑	Akt/mTOR pathway	[23]

Cucurbitacin B	Pressure overload- induced cardiac hypertrophy	AB-induced mice	Autophagy ↑	Heart weight, cross-sectional area Interstitial fibrosis ↓ Systolic and diastolic ↑ Normalized in gene expression Reserved microvascular density	Akt/mTOR/FoxO3a signal axis	[24]

Trimetazidine	Diabetic cardiomyopathy	Type 2 DCM rat	Autophagy ↑	CVF, PVCA/LA ratio, phosphorylation of ERK and P38 MAPK ↓ Phosphorylation of AMPK, the interaction between Bcl-2 and Beclin-1 ↑	PI3K/AKT and AMPK pathways	[25]

Liraglutide	Diabetic cardiomyopathy	ZDF rat	Autophagy ↑	CK, LDH, mTOR phosphorylation ↓ AMPK phosphorylation ↑	AMPK-mTOR signaling pathway	[26]

1,25-Dihydroxyvitamin-D3	Diabetic cardiomyopathy	STZ-induced type 1 diabetic rat High-glucose cultured H9C2 cells	Autophagy ↑	Myocardial hypertrophy and interstitial fibrosis ↓ Cardiac function ↑	*β*-Catenin/TCF4/GSK-3*β* and mTOR pathway	[27]

Spermidine	Physiological cardiac aging	Wild-type C57BL/6 mice	Autophagy ↑	LC3-II, autophagic flux ↑ Cardiac hypertrophy ↓ Preserved diastolic function		[28]

Isoflurane	Cardiomyocytes from A/R injury	Cardiomyocytes from rat	Autophagy ↓	Cell viability ↑ Autophagosome generation, expression of NOD2 ↓	P38MAPK pathway	[29]

Aliskiren	Pressure overload-induced heart hypertrophy and fibrosis	TAC mice	Autophagy ↓	Expression of Atg5 and Atg16 L1 mRNAs, LC3-II and Beclin-1 proteins ↓	Ang II-PKC*β*I-ERK1/2-regulated autophagy	[30]

Calhex 231	Cardiac hypertrophy	TAC or Ang II-induced rat	Autophagy ↓	CaSR expression, autophagy levels ↓	CaMKK*β*-AMPK-mTOR pathway	[31]

Hydrogen sulfide	Diabetic myocardial	STZ-induced diabetic rat	Autophagy ↓	Myocardial fibrosis, myocardial autophagy ↓ PI3K/AKT1 pathway ↑	PI3K/AKT1 signaling pathway	[32]

Chloroquine	Diabetic cardiomyopathy.	STZ-induced diabetic mice	Autophagy ↓	Ameliorated cardiac diastolic function autophagolysosomes, cardiomyocyte apoptosis, cardiac fibrosis ↓ LC3-II, p62 expression ↑	mTOR pathway	[33]

Multi-Strain Probiotics	Obesity-induced cardiac fibrosis	HF diet-induced obese rat	Autophagy ↓	TGF/MMP2/MMP9 fibrosis pathways, ERK5/uPA/ANP cardiac hypertrophy pathways ↓ Beclin-1/LC3B/Atg7 pathway ↑	TGF/MMP2/MMP9 fibrosis pathways, ERK5/uPA/ANP cardiac hypertrophy pathways, Beclin-1/LC3B/Atg7 autophagy pathway	[34]

Phellinus linteus Mycelium	Myocardial I/R Injury	I/R rat	Autophagy balance	Infarct size, plasma lactate dehydrogenase level, caspase-3, caspase-9, Beclin-1, p62, mTOR level ↓ Bcl-2/Bax ratio, AMPK Phosphorylation ↑	AMPK-dependent pathway, Beclin-1-dependent pathway	[35]

*Note.* AMI, acute myocardial infarction; AMPK, AMP-activated protein kinase; mTOR, mammalian target of rapamycin; SHRs, spontaneously hypertensive rats; AB, aortic banding; CVF, collagen volume fraction; ERK, extracellular regulated protein kinases; MAPK, mitogen-activated protein kinases; DCM, diabetic cardiomyopathy; ZDF, Zucker diabetic fatty; CK, creatine kinase; LDH, lactate dehydrogenase; STZ, streptozotocin; A/R, anoxia/reoxygenation; NOD2, nucleotide binding oligomerization domain containing 2; TAC, transverse aortic constriction; Atg, autophagy-related gene; ATG16 L1, autophagy-related 16-like 1; LC3-II, Light chain 3-II; P38 MAPK, P38 mitogen-activated protein kinase; ERK, extracellular signal regulated kinase; PKC, protein kinase C; Ang II, angiotensin II; CaMKK *β*, Ca 2+/calmodulin-dependent-protein kinase-kinase-*β*; CaSR, calcium-sensing receptor; PI3K, phosphatidylinositol-4,5-bisphosphate 3-kinase; HF, high-fat; TGF, transforming growth factor; MMP2, matrix metalloproteinase-2; MMP9, matrix metalloproteinase-9; I/R, ischemia-reperfusion.

## Abbreviations

MF: Myocardial fibrosis
DCM: Diabetic cardiomyopathy
RAAS: Rennin-angiotensin-aldosterone system
I/R: Ischemia/reperfusion
mTORC1: Mammalian target of rapamycin complex 1
ULK: Unc-51-Like Kinase
FIP200: FAK-Family Interacting Protein of 200 kDa
Atg13: Autophagy-related Protein 13
AMBRA1: Activating Molecule in Beclin-1-Regulated Autophagy
PI3K: Phosphatidylinositol-3-kinase
VPS15: Vacuolar Protein Sorting 15
Ubl: Ubiquitin-like
ESCRT: Endosomal sorting complex required for transport
LC: Light chain
LVH: Left ventricular hypertrophy
3-MA: 3-Methyladenine
TAC: Transverse aortic contraction
ERK1/2: Extracellular signal-regulated kinase
MAPK: Mitogen-activated protein kinase
AT1R: Angiotensin II type 1 receptor
PKD: Protein kinase D
Ang II: Angiotensin II
CREG: Cellular repressor of E1A-stimulated genes
bFGF: Basic fibroblast growth factor
OGD: Oxygen-glucose deprivation
1,25D3: 1,25-Dihydroxyvitamin-D3
AMPK: AMP-activated protein kinase
PINK1: PTEN induced putative kinase 1
AMI: Myocardial infarction
AB: Aortic banding
TCF: T-cell factor
GSK-3*β*: Glycogen synthase kinase-3*β*
NOD2: Nucleotide binding oligomerization domain containing 2.
